# Supporting and enabling health research in a local authority (SERLA): an exploratory study

**DOI:** 10.1186/s12889-022-13396-2

**Published:** 2022-07-09

**Authors:** Ciara E. McGee, Megan Barlow-Pay, Ivaylo Vassilev, Janis Baird, Lee-Ann Fenge, Debbie Chase, Julie Parkes

**Affiliations:** 1 National Institute for Health Research, Clinical Research Network Wessex, Southampton, UK; 2 Public Health, Southampton City Council, UK; 3 National Institute for Health Research, Research Design Service South Central, Southampton, UK; 4 Health Sciences, Environmental and Life Sciences, University of Southampton, Southampton, UK; 5 MRC Lifecourse Epidemiology Centre, University of Southampton, Southampton, UK; 6 Southampton Biomedical Research Centre, National Institute for Health Research, University of Southampton, University Hospital Southampton NHS Foundation Trust, Southampton, UK; 7 Faculty of Health and Social Sciences, Bournemouth University, Poole, UK; 8 School of Primary Care, Population Sciences and Medical Education, University of Southampton, Southampton, UK; 9 Applied Research Collaboration Wessex, National Institute for Health Research, Southampton, UK

**Keywords:** Public health, Local authority, Research

## Abstract

**Background:**

The use of research evidence to underpin public health practice and policy decisions in local government is strongly promoted but its implementation has not been straightforward. This study aimed to explore the factors, relationships and processes that contribute towards accessing, using, and generating research evidence that is relevant to local authority public health and social care and shapes its practice.

**Methods:**

Semi-structured individual interviews with elected councillors, officers directly involved with public health and social care and with community members from one urban unitary authority in South England were conducted. Interviews were audio recorded, transcribed verbatim and thematically analysed.

**Results:**

Fourteen participants took part in the semi-structured interviews. Local knowledge and evidence are prioritised, and anecdotal evidence is valued. The Director of Public Health was the principal source of information and support. Academics were rarely mentioned as information sources, and their involvement was ad hoc. The use of research evidence varied between individuals and departments, with wider engagement among public health specialists. Key barriers to the use of research evidence included access (not reported among public health professionals), research timeliness, local applicability, competence in finding and interpreting evidence and the role of research evidence within a political context. Public health and adult social care teams are not currently research active or research ready. Major barriers exist due to financial constraints and the socio-political context of local authorities. COVID-19 disrupted siloed ways of working, strengthening and opening potential collaborations within the local authority. This changed perspectives about the value of research but is likely time-limited unless underpinned by sustainable funding.

**Conclusion:**

Creating strategic level roles within local government to work with the Director of Public Health to champion the research agenda and embedding researchers within and across teams would build capacity for local authorities to sustainably co-create, undertake, and use evidence to better inform future actions.

## Background

In 2013, the responsibility for public health was transferred back to local authorities in England as part of the Government’s health and social care reforms [1]. Local authorities were given legal responsibility to improve local public health and reduce health inequalities [2], with elected councillors inheriting ultimate decision-making powers for public health priority setting and commissioning [2]. Local authority public health is a legal duty underpinned by an annual public health grant from the Department of Health and Social Care. In 2019/20 the total public health grant for local authorities was £3.13 billion [3] but this grant has been significantly cut by £200 million in recent years [4]. Continuous cuts to public services pose significant challenges for local authorities, increasing the need for reliable and timely evidence to optimise the use of resources to improve population health [5].

Using research evidence to improve public health outcomes is widely recommended as it supports decision and policy maker understanding by framing options and addressing implementation considerations [6]. Academics seek to produce usable evidence to inform public health practice [7] but a recent systematic scoping review found that research evidence is underutilised in local authorities because it is not always relevant to the local context due to its more global nature [8]. Consequently, decision makers may turn to other sources, such as expert opinion, anecdotal information, and local intelligence and evaluations of unknown quality [9]. Barriers to the use of research evidence include time constraints, capacity and expertise, mismatched timescales between  policy and academic research, access to and availability of research, and the role of research evidence within a political organisation [9–13]. Nevertheless, there is an appetite for using research evidence to inform public health practice and decision making which demonstrates local salience [9–11].

Current literature postulates that researchers need to understand and respond to the local priority evidence needs of public health decision makers [8–11]. Co-production between academia and local authorities through joint appointments and/or embedding researchers in the local authority may support more meaningful outcomes but flexible research funding is needed to support such models [14, 15]. The Academy of Medical Sciences calls for transdisciplinary research to tackle future public health challenges [16] and involving local authorities in the creation of public health evidence may better inform local public health actions [17].

The National Institute for Health Research (NIHR) have a long history of providing significant funding for National Health Service (NHS) research and research infrastructure, including developing a skilled workforce, pledging to extend support into non-NHS public health and social care sectors [18]. Building an evidence-base for local authority public health is required to improve the health of the public and reduce inequalities in health [6]. It has been recommended that the NIHR provide relevant mirroring of NHS research infrastructure for non-NHS environments to inform public health actions and meet the needs of the population [17] but local authorities are complex systems with internal (staff, structures, cultural values) and external (political environment, national directive) influences [19] and this presents challenges. To our knowledge there is no published research evidence about how a research system could be developed, operationalised, and maintained within a political organisation. This study therefore used two major public health challenges as exemplars to provide current and credible insight into the public health actions in relation to standard practice (childhood obesity) and a crisis situation (Covid-19), and identify the resources needed to support and enable research within a local authority environment.

Childhood obesity and the current COVID-19 crisis were chosen exemplars for this study since both were a high priority within the local authority. Childhood obesity is one of the public health priorities for the local authority under study and has cross party-political support as demonstrated in its recent scrutiny inquiry into childhood obesity. The local authority was proactively involved in planning and preparing action to protect its community from the spread of COVID-19. The authors felt that including these two areas provided a comparison between an on-going public health concern and an emergency public health problem was likely to illuminate some of the mechanisms that lead to closer engagement.

 Using the long-term challenge of childhood obesity and the evolving COVID-19 pandemic as exemplars this study aimed to develop an understanding of the factors, relationships and processes that contribute towards accessing, using, and generating research evidence that is relevant to local authority public health and social care and shapes its practice.

Key objectives of the study were to:

- 1. Understand the sources of information and evidence used in the local authority response to tackle childhood obesity and COVID-19.
- 2. Explore the attitudes and perceptions of research and evaluation among elected members and officers directly involved with public health and social care.
- 3. Develop an understanding about what is necessary to create a research system to sustainably develop influential and innovative research activity within the local authority.
- 4. Explore how community groups and the public are currently involved with council services and their views about how processes can be developed to embed greater involvement in future research and evaluation.

## Methods

### Participants and recruitment

Semi-structured individual interviews were conducted in one urban unitary local authority in South England with elected councillors and officers directly involved with public health and adult social care and with community members. The local authority was conveniently selected for this study based on existing professional networks and having an embedded researcher (CM) within the public health team. The Director of Public Health identified potential participants whose work related to public health and social care. Community participants were identified via Patient and Public Involvement (PPI) representatives and represent a range of demographics, particularly communities experiencing health inequalities and poor health outcomes. Seventeen potential participants were invited to take part in the interviews via email with fourteen (*n* = 1 withdrew and *n* = 2 did not respond) agreeing to participate. Potential participants were invited to take part in a semi-structured interview via email with an enclosed information sheet and consent form. Informed consent was obtained from all participants in the study. Community participants received a £20 voucher for participation in the study.

#### Interviews

An interview guide was developed covering topics relevant to the study research aims and objectives. During the interviews, to facilitate exploration of the sources of information used by participants we used GENIE [20], an online social networking tool to map the relationships and or connections between people and organisations which showed where and from whom information was sought. A separate interview guide was co-developed for community representatives in collaboration with PPI representatives. Following a pilot test of the interview guide [21] with one Public Health consultant not involved with the study, we revised the interview schedule to maximise clarity of questions and pace the interview. Due to the COVID-19 pandemic and social distancing, audio-recorded interviews with participant consent were conducted using Microsoft Teams (*n* = 11) and over the telephone (*n* = 3) between September and October 2020 and lasted between 40 and 60 min. Interviews with council participants and community members were conducted by CM and MB, respectively. At the time of the study the researcher and first author (CM) was embedded within the public health team and was therefore known to some (*n* = 6) of the study participants.

#### Data analysis

Interview audio recordings were transcribed verbatim, entered in NVivo 12 software, and thematically analysed [22]. All interview transcripts were read and re-read with the council interviews and community interviews being coded by CM and MB, respectively. Interviewers undertook initial coding using a combination of inductive and deductive techniques to generate codes. Broad codes were collapsed into higher and lower order themes to develop descriptive and interpretive summaries. To aid credibility and trustworthiness of findings, analyses and interpretation were discussed and checked with the research team. Community interview analysis and interpretation went through a process of consultation with PPI representatives.

## Results

Findings from the local authority and community interviews are presented and discussed separately with a wide range of anonymised quotations under thematic categories. The findings provide an insight into the processes and practices for generating and using evidence to address local concerns, while also highlighting the different sets of complexities experienced within local authorities and communities.

### Findings from the local authority interviews

We present a broad range of key themes from the local authority interviews which highlight some of the key tensions and processes involved in the negotiating between different groups of professionals within the local authority, particularly in relation to political agendas and political processes and the generation and use of evidence.

#### The childhood obesity and COVID-19 response

Childhood obesity is an important public health and government priority, but most study participants focused on COVID-19 as a priority response due to its immediate threat, and prioritisation nationally and locally. Few participants were directly involved in the childhood obesity response, aside from the Director of Public Health, a Senior Public Health Practitioner, and the Ward Councillor on the Scrutiny Panel. A recent scrutiny inquiry, led by a panel of elected councillors, reviewed a range of stakeholder and expert witness responses to how evidence shaped the council response to tackling childhood obesity. The inquiry also raised awareness of the issue of childhood obesity among other departments and elected members, highlighting the need for a long-term commitment and willingness for flexibility across council functions, and with partners, including the Government.

*“What I hope will come out of the scrutiny is more joined up working between the city council and the university, more understanding of the drivers on both sides, accepting what needs to be done in an academic sense… but to say that sometimes this may not result in a Lancet publication, but if it helps the delivery of services or stopping services that are not working or helps us to evaluate. So, I hope that because of the juxtaposition of the university and me being a councillor and having that overlap we may be able to... take advantage of the expertise of how to do proper research.”*

Participant responses demonstrated that COVID-19 responses were strategic, with the development of the Local Outbreak Control Plan, and delivery of the plan through a comprehensive programme of projects including testing, contact tracing, intelligence, communications, community engagement via COVID-19 Community Champions, and Public Health information-cell provision. Consultants in Public Health provided strategic and operational support under the leadership of the Director of Public Health, and key Officers from across the local authority were tasked with leading projects to support a whole council response. COVID-19 has led to a move away from operational silos, increasing partnership working for example, between Public Health and Adult Social Care to support with personal protective equipment  procurement, and the prevention and management of COVID-19 outbreaks within care homes. Social Care professionals commended public health guidance and support which enhanced their response.

*“Adult social care had some connect points with public health, but never like this… They [public health] gave us the information and data, the likely trajectory and what was going to happen, the number of people that you were going to see within hospitals and the impact on local communities, which enabled us to move our resources to the most appropriate places. That was very key”.*

#### Sources of information utilised in the childhood obesity and COVID-19 response

During the interviews participants were asked about the main sources of information they used to inform the local authority response to childhood obesity and COVID-19. We used GENIE (as described in the methods) to help with this. The Director of Public Health was the most frequently cited source of information and support, alongside other local authority staff and elected members. Outside the local authority, local networks of Directors of Public Health, the Association of Directors of Adult Social Services, the Local Resilience Forum, and the Clinical Commissioning Group were cited as sources of information in the COVID-19 response, alongside senior academic involvement in the COVID-19 Health Protection Board and COVID-19 Saliva Testing Programme. Childhood obesity networks included the 0–19 years partnership and practitioner forums, and links with universities via academic presentations at the childhood obesity scrutiny inquiry. Academic research did not explicitly feature during the network mapping exercise but was accessed through National Institute of Clinical Excellence Guidance, Public Health England, Office for National Statistics, Local Government Association bulletins and government websites. Participants highlighted that understanding and accessing evidence, and its interpretation and contextualisation alongside experiential knowledge and peer learning was treated and experienced as an individual responsibility.

“In terms of understanding the evidence, I would say that is our role to understand the evidence.”

“The ADASS [Association of Directors of Adult Social Services], you need your own networks to help inform decisions and conversations, so if you’ve got queries, you can put that out to that wider group.”

“I'm in a network of Directors of Public Health and we come together on a weekly basis… that’s important from a peer support perspective.”

#### Multiple concepts, preferences, and priorities of evidence

Participants provided broad definitions of what counts as evidence and its use within a local authority context.

*“It’s looking at international and national evidence. The other type of evidence is data. We run city surveys to understand what the public are doing, what they are thinking, how they are interacting.”*

*“If it’s evidence it’s data, or it could be anecdotal but gathered in a meaningful way that demonstrates how residents are feeling or demonstrates the impact of an intervention or an activity.”*

Emphasis was placed on quantitative data, primarily using descriptive statistics rather than qualitative sources, although a lack of coherent strategy across departments on evidence use lead to frustration.

*“I couldn't say hand-on-heart that every single council service does that well. There are some that are doing it well like the integrated commissioning unit because they straddle the health service and the council operations. Adult social care I'd say are doing it to a degree, but it could be strengthened, and public health do it…. that is part of our culture.”*

*“If I’m absolutely honest, with our* local authority *I would say that we don’t’ have one version of the truth. We have lots of different versions… everyone’s got a different version of what their evidence is.”*

Some councillors preferred anecdotal evidence from constituents, but local government officers are perceived as having responsibility to present evidence to support councillor decision-making.

*“Each individual councillor will look at evidence very differently. There are some who will only work anecdotally.”*

*“It’s not necessarily the priority of the councillor to look at the evidence, its incumbent on the persons who is championing that topic to make sure that they present the evidence in a way that resonates with the councillor.”*

Priority of evidence differed across local authorities and NHS organisations, and local authorities exist within a wider socio-political context requiring a balance between evidence within these wider requirements.

*“In the NHS, it is almost easier because you’re in a health environment and you’re thinking, what is this health intervention? What does the evidence show us…? When you work in a political environment, you're balancing that evidence with a political approach to support society for a population.”*

#### Types of evidence used

A wide range of evidence was accessed including online and local data, peers, networks, and social media to keep abreast of emerging COVID-19 evidence. Few accessed academic research evidence directly even though public health participants have free open access to scientific journal databases via visitor status at one local University. Participants from adult social care relied on other sources to collate and synthesise available research evidence including the Social Care Institute for Excellence and Research in Practice for Adults, which triangulates academic research, practice expertise, and service user insights. Triangulating evidence was seen as integral for social care and public health to a create a rich and relevant knowledge base.

#### Barriers to and facilitators for evidence-informed practice

Barriers for evidence use related to access (except for public health participants) and accessibility; time and timeliness of research evidence; the political process; lack of relevant research applicable to the local context; and competence to find, analyse and interpret research evidence (see Table 1). Public health participants require timely evidence that is relevant to their real-world practice settings, as academic research often lags behind urgent decision-making processes.

*“We need to make decisions quickly… Public health intervention doesn't fit an evidence-based model well because of this whole system approach that's needed… With childhood obesity we have got great evidence that you can do pockets of things, but what we don’t have and need more of is evidence of whole systems approaches.”*

**Table 1:** Barriers to evidence use in the local authority

Theme	*Quote*
Access and accessibility	*“The question is how would be get access to [academic research] and how accessible is it for the non-scientists among us?”*
Time constraints	*“The key one is time… people have different time pressures.”*
Timeliness of research	*“Sometimes people like Public Health England collate and rapidly pull evidence together and that is helpful, but often it takes a long time to do it. You [Public Health] want it now. They [academics] are going to bring research in six months or next year… so there is something about the timeliness of evidence.”*
Political processes	*“I don’t feel like we [politicians] are often making good decisions based on good evidence. I think a lot of the decision making is driven by will it look good, can we put it on a leaflet that we can go out and get people to vote for us, because the primary driver for politicians is to stay in power.”*
Research relevance	*“I think there’s something about pragmatism… so we can look at the most thorough trials… but can you apply that to our local context, given our population needs? I think that’s challenging.”*
Competencies	*“I don’t feel very skilled to be able to look for research evidence and interpreting some of it because I don’t regularly do that… I would definitely need some training.”*

Social care participants highlighted how COVID-19 increased pressure on adult social care services and the need for evidence to forecast future demands.

*“There's a huge amount of work that we need to do collectively in the system to properly understand the impact of COVID. I want a demand model for adult social care, based on the health of the population and what that means... There’s a need to properly consider the impact of COVID and how we measure that.”*

Training was identified as one solution for improving competencies and overcoming ‘*fears*’ of using evidence, particularly in adult social care to support engagement with a range of evidence including grey literature, practice guidance, service user perspectives and academic research.

*“It’s breaking down barriers of fear around evidence because it feels scary… My assumption is that it’s really difficult to do, to look for research, I don’t really know where to go to or how to do it and then what to do with it.”*

*“What [University name] have is an arrangement that all Public Health teams in the South West can attend modules for free. It's not that they [practitioners] don't want to have those skills, it's just they haven't necessarily had the opportunity.”*

#### Barriers to engaging with research

There was much enthusiasm for local authority involvement in generating public health and social care research and evaluation. However, teams are currently not research active or research ready. Major barriers exist due to the socio-political context of local authorities. Research is just one source of information for policy makers, and engagement is restricted due to limited resources, organisational capacity, local priorities, time constraints and short political cycles.

*“I see that [research] as a great opportunity. Particularly with COVID, I have seen a shift in understanding the value and importance of an evidence-based approach… Money, resources, that’s the massive challenge. It is the elephant in the room.* *have been cut right back.”*

*“It’s a question of how we make sure it [research] is relevant to what we want to do and the pressure that colleagues might feel about it. I think that’s one of the challenges of [political] short-termism.”*

#### Supporting and enabling research capacity

Participants suggested that local authority research could boost investment and funding to challenge the “*Cinderella Service*” mindset. Participants postulated COVID-19 presented an opportunity to establish a research climate within the local authority but unless supported by additional resources this is likely time-limited.

*“If there was a greater body of research in local authorities, then that might get properly resourced, funded, and looked at in different way rather than just it’s a bit of Cinderella service.”*

*“Particularly with COVID, the time is right to set-up that culture, the policies to enable us to do that. The problem is people. Its resources to support it, and without that I’m afraid it won’t happen.”*

Su﻿ggested solutions for supporting research capacity within the local authority included recruiting people with skills in research methodology, data analysis and evaluation to better inform practice and decisions.

*“I would recruit people skilled in research and evaluation and embed them in teams.”*

“*We need methodologists to take us with them. So, they lead and inform and support us in the methods and approaches we take within our local authorities.”*

Developing academic support to formulate research questions, alongside co-production with diverse communities, would enable more effective local community responses.

*“There’s a host of questions that as a public health consultant we have but turning that into research question is a different thing… so I need a university to help pull that out.”*

*“There are particular groups of the community and vulnerable people that we need to pay particular attention to and do things slightly different for and its thinking about who those groups might be…. It’s about ensuring we have a two-way process with the community.”*

Strategic development of appropriate infrastructure for local authority research activity, alongside leadership support and commitment for research, are crucial.

*“My suggestion is to take someone at the level of [person’s name] from the NIHR and they work a day a week working through what this infrastructure looks like and what resources would be made available to better support us.”*

*“I’d like to think this structure that we’re developing secures from the local authority leaders itself a commitment to research and evaluation... there needs to be supportive structures from leadership, as well as operationally.”*

Councillors echoed this through recognition of the importance of linking research to council priorities to reinforce the value of research to the administration.

*“Saying [to politicians] I can see this is what you want to do. We could evaluate that, and that evaluation could lead to it being made bigger, being rolled out across the country… That’s how you get politicians to understand research.”*

#### Promoting and improving academic relationships and collaborations

The value of collaborative relationships with universities was recognised but these were seen as opportunistic rather than strategic. Major barriers to forming and maintaining academic links relate to timing, differences in thinking, financial costs of academic involvement, and knowing who to connect with.

“They’ve stalled because of timing or a slight mismatch between what local authority people are thinking and what academic people are thinking…”*“They’ve stalled because of timing or a slight mismatch between what local authority people are thinking and what academic people are thinking…”*

*“I know there are universities, but I don’t know how you would make those initial links. I don’t know how to develop that relationship with an evidence organisation.”*

Wider academic networks across higher education institutions are needed to strengthen support for local authorities with improved communication around culture and priorities to identify mutual interests and research opportunities.

*“The Wessex forum supports us to create that link with the University. I’d argue we’re not doing that well enough across all Universities… there is a need for Universities to come together in their own network, so they can strengthen what that academic offer is.”*

*“I think it would be helpful for universities to have an appreciation of the political environment… then they would be able to work out how to influence local authorities and get that research.”*

*“I guess universities just letting local authorities know what research they are doing and giving them the opportunity to get involved... having discussions about what would be helpful for both sides.”*

Embedded researchers, joint appointments, and models of support between the local authority and academia can bridge the gap between research and practice.

*“Having that embedded researcher role has been crucial in keeping this going. You are almost like a little bit of thread holding us together... we need to strengthen that link.”*

*“They [researchers] could be the leads in terms of stakeholder relationships with universities and making connections and bridging the gap between research and practice. A joint post would be a lovely way of doing it.”*

Social work teaching partnerships offer an example of local authority and higher education institution collaborations and a step towards improving evidence-based practice. Working within practice can inform teaching about public health issues, and local authority student placements provide a balance between theoretical knowledge and real-world problems.

*“I can bring real-life stuff to them [students]… I would love to get to a stage where we are utilising students and getting them into work that is really needed… I think there would be huge opportunities there.”*

### Findings from the community interviews

We present two broad themes from the community interviews which identified the need for meaningful and sustainable engagement with local communities, and their capabilities as well as their priorities and needs.

#### Negotiating the meaning of community, representation, and involvement

Different ways of engaging with the local authority include through tenant’s panels, although the effectiveness of these were questioned.

*“I think you should bear in mind is that I reckon that the average age of people on tenant panels is going to be over 60 because they're the only people that have got the time.”*

One participant, representing a local mosque, spoke about council members attending the mosque to share information.

 "Well, when we opened up the centre, I remember lots of council used to come because we invited the mayor and others to open up different events." 

All community interviewees were active and engaged members of their community but were not considered representative of the groups they represent (e.g., residents of a social housing estate) and activity is largely driven by a few motivated individuals.

"*What a lot of people have got to realise is unfortunately a lot of people don't want to do anything for their community, they rely on people like me.”*

*“It does depend on how it's presented. I find it very difficult because I've made a conscious decision to get involved in whatever it might be… there’s only one way to change it, and that's to get involved, not sit back and moan and complain.”*

It was considered challenging to engage wider community members with activities which require longer-term commitments.

*“When they [the council] set up panels, okay, they said that nobody should stay on a panel for more than five years... because they believe that they will be inundated with people…. it has not happened like that. You're not getting enough people on the panels.”*

A mosque volunteer recommended trying more ad hoc flexible approaches of engagement in spaces already attended (in this case at Friday prayers).

*“Oh yes, they will talk about things, but if you want them to participate to write things out or to join something or do something, that's not that easy. On a Friday, for example, if you come and you want to do quick research by questionnaire type of thing… but to do something substantial, yes, they usually switch off.”*

#### Understanding the process of community (dis)engagement

Community interviews indicated that a lack of interest and or ability to sign up to current long-term models (such as panels) for involvement were a barrier for those with competing prioritised or chaotic lives. Therefore, a combination of longer-term relationship building and co-production methods, alongside the opportunity to input and collaborate informally was recommended. Barriers of community engagement were often compounded by cultural or language barriers which may deter people from Black, Asian, and Minority Ethnic groups and disproportionately affect women from some communities. Barriers can be systemic resulting from a lack of understanding or bias by researchers about community needs.

*“Sometimes it's imagined barriers. You might think oh I am English, or I am something, if I go there, I have to be this, that or maybe they don't like. It is not like that; you should try to come and participate now. I'm not just talking about the Muslim community, go into other communities as well.”*

The use of incentives could motivate people to take part in research, although financial remuneration should not affect benefit entitlement. Vouchers, small gifts, thank you cards, and recognition may incentivise involvement. Preventing logistical barriers is important, including using accessible locations or providing transport.

*“You were never going to get people to go out unless you can arrange transit. That would be an incentive, and then financial incentive or a voucher for whatever.”*

All participants positively referenced projects undertaken in communities by Non-Governmental Organisations and community groups, highlighting the importance of local knowledge and existing relationships and trust. Religious organisations engage with a wide range of external stakeholders and their networks offer access opportunities to different people.

Participants appeared motivated by issues which directly affected them, or their community. Health and social care research were considered something that happens ‘*somewhere else’* resulting in a lack of understanding. For example, one participant described how a previous research group that tried to recruit through their community but struggled with recruitment as it was perceived too complicated and not directly relevant.

## Discussion

This paper presents the findings from semi-structured interviews with local government elected councillors, council officers and community members from one urban region in South England. Our findings demonstrated that research and evaluation are not a high priority for local government and budgets cuts contribute to a lack of research capacity. Enthusiasm for local authority research exists in principle and interviews highlight the value of research evidence to inform practice. However, barriers include time constraints, lack of resources and capacity, organisational priorities, and short political cycles. These findings concur with previous studies highlighting barriers to evidence use, including access (not reported among public health participants), timeliness of research evidence, and competence in finding and appraising evidence and the political context of the local authority [6, 8–10]. Multiple perspectives on what counts as evidence exist; local knowledge and evidence is prioritised, and anecdotal evidence is valued [23]. Evidence use varied between individuals and departments, with wider engagement among public health specialists reflecting an existing research culture [23]. COVID-19 disrupted siloed ways of working, strengthening and opening potential collaborations within the local authority. This changed perspectives about the value of research but is likely time-limited unless underpinned by sustainable funding. Additionally, access to scientific publications and confidence in using scientific databases by local authority staff are unlikely to improve the use of evidence without additional support. Even where such access was available it was sources such as the National Institute for Health and Care Excellence Guidance, Public Health England, Office for National Statistics, government websites and experts in the area, principally the Director of Public Health, that were prioritised sources of information and support. Academics were rarely mentioned as information sources [24] and their involvement was mostly ad hoc or through invitation to specific forums.

Our findings suggest a need to commit at a strategic level to joint appointments and new research roles embedded within the local authorities. Such appointments may help make the use of research evidence normal practice in local authorities, support staff to use existing evidence in a manner that is realistic and adds value to their work and help build sustainable links with existing research infrastructure. This might be through for example their understanding of existing practice in both contexts such as identifying projects with mutual local benefit. Further research is required to explore sustainability and how this would work for different contexts and role priorities. Collaborative approaches such as the co-production of relevant and timely research between academic researchers and local government staff has long been recommended to increase the use of evidence in local government practice and decision-making [25, 26]. Collaborative models such as the use of embedded researchers in practice have shown promise for strengthening networks and creating meaningful engagement between local authority public health and academic researchers in the co-production of evidence [14]. However, embedding research and strengthening the uptake of evidence use in local government requires senior level buy-in and commitment, as leaders set the tone for organisational climate and are a key facilitator of capacity building and practice [27, 28]. Closer interaction and engagement between elected members, chief officers and researchers could facilitate a culture of research and evidence use by providing more mutual understanding of the structures and challenges under which local government staff and academic researchers work [28, 29]. Many authors recommend new governance arrangements in the form of collective reflective spaces that facilitate co-production of new knowledge between government departments and external partners [30].

Interviews with community participants highlighted several challenges for engaging diverse communities with council services and suggested facilitators for embedding greater community involvement in future research. Much activity was perceived to be driven by too few key motivated individuals. Barriers to wider community involvement include structures requiring too much commitment, the timing and location of meetings resulting in less diversity of representation. Bias and assumptions by researchers may compound lack of engagement due to perceived difficulties in accessing seldom heard groups. Developing enthusiasm and commitment was considered key if research is to be seen as meaningful by community participants. Providing relevant, clear, and meaningful feedback may improve engagement, alongside flexible ad hoc opportunities, and incentives for involvement.

## Strengths and limitations

The strength of this study includes having an embedded researcher within the public health team and our existing academic relationships with the Director of Public Health which has enabled us to conduct this study in the middle of a pandemic and gather a range of representative views. Moreover, this study was part of the NIHR preliminary drive to better understand the resources needed to support and enable research activity within local government and is sited in a body of similar research and contributes to this evidence base. The study also has some limitations as it was based on a small number of interviews from one urban unitary authority in South England and some findings may not be generalisable to local authorities in other regions. The local authority under study is a medium-sized unitary authority within the worst national deprivation quintile, and experiences significant and persistent health inequalities and thus different priorities to other regions. Data gathered from local Councillors were the opinions of one political party and may not represent other opinions. Despite efforts to limit methodological bias, results are based on qualitative research which is by nature subjective. The study was conducted during a pandemic and over a short time frame which may have led to possible restriction in numbers of available participants, with some being unable to take part in the study due to competing priorities. Despite these limitations, a range of views are represented, and it is unlikely that these limitations compromise the integrity of the study.

## Conclusions and recommendations

There is appetite for local authority public health and adult social care research and although COVID-19 is an unforeseen national public health emergency that has caused significant disruption it has also created a catalyst to strengthen a culture of research and evidence use in within the local authority. However, findings highlight the challenges faced by local authority public health and adult social care teams wanting to co-create, undertake, and use research evidence to inform future actions. Adequate support and sustainable funding for research and research infrastructure is required to address these challenges to enable and embed public health and social care research within local government. Based on our participants’ suggestions for supporting and embedding research within local government and greater community involvement in future research processes several recommendations for consideration are listed in Tables 2 and 3 below.

**Table 2:** Recommendations to support and enable research within local government

• Embed strategic level appointments within the public team to work with the Director of Public Health to champion the research agenda and value of research and build links to existing research infrastructure. This could include joint appointments across higher education institutions and the local authority
• Embed researchers within the local authority to (I) aid the delivery of meaningful evidence within local authorities (II) help staff to work with existing data (III) upskill staff through short courses and one-to-one tutoring (e.g. evidence synthesis; rapid reviews) (IV) facilitate the generation of local research and evaluation by supporting local authority staff (V) expand links across teams within the local authority and (VI) support the local authority to develop partnerships with higher education institutions working on common projects and or funding bids
• Create an open data repository platform to bring together local authority public health, social care, and other local authority research evidence to eliminate duplication to create holistic approaches for tackling population health needs. Encourage local authority staff to use the platform to generate research questions and ideas of relevance to local authority and engage the embedded researcher(s) and strategic research lead to explore potential links to academics in higher education institutions

**Table 3:** Recommendations to embed greater community involvement in future research

• Harness existing community knowledge by building relationships with individuals and NGOs already working in the community
• Consider communities as ‘under-represented’ rather than ‘hard to reach’ to counteract systemic barriers. Explore incentives for involvement
• Ensure PPI involvement in public health and social care research is presented in relevant and meaningful ways to reflect individuals’ lived experiences
• Explore inclusive modes of involvement, with appropriate training and feedback on involvement

## Data Availability

Transcripts that support the findings of this study are not publicly available but anonymised data are available from the corresponding author upon reasonable request.

## Abbreviations

NIHR: National Institute for Health Research
NHS: National Health Service
PPI: Patient and Public Involvement

## References

[1] Department of Public Health (2011). Public Health in Local Government.

[2] Gorsky M, Lock K, Hogarth S (2014). Public health and English local government: historical perspectives on the impact of 'returning home'. J Public Health (Oxf).

[3] Department of Health and Social Care (2018). Public health ring-fenced grang 2019/20 circular.

[4] TheKing'sFund (2017). Chickens coming home to roost: local government public health budgets for 2017/18.

[5] Dorling H, Cook A, Ollerhead L, Westmore M (2015). The NIHR Public Health Research Programme: responding to local authority research needs in the United Kingdom. Health Res Policy Syst.

[6] Brownson RC, Fielding JE, Maylahn CM (2009). Evidence-based public health: a fundamental concept for public health practice. Annu Rev Public Health.

[7] Whitty CJ (2015). What makes an academic paper useful for health policy?. BMC Med.

[8] Kneale D, Rojas-García A, Raine R, Thomas J (2017). The use of evidence in English local public health decision-making: a systematic scoping review. Implement Sci.

[9] Kneale D, Rojas-García A, Thomas J (2019). Obstacles and opportunities to using research evidence in local public health decision-making in England. Health Res Policy Syst.

[10] Atkins L, Kelly MP, Littleford C, Leng G, Michie S (2017). Reversing the pipeline? Implementing public health evidence-based guidance in english local government. Implement Sci.

[11] Curtis K, Fulton E, Brown K (2018). Factors influencing application of behavioural science evidence by public health decision-makers and practitioners, and implications for practice. Prev Med Rep.

[12] Brownson RC, Fielding JE, Green LW (2018). Building Capacity for Evidence-Based Public Health: Reconciling the Pulls of Practice and the Push of Research. Annu Rev Public Health.

[13] Orton L, Lloyd-Williams F, Taylor-Robinson D, O'Flaherty M, Capewell S (2011). The use of research evidence in public health decision making processes: systematic review. PLoS ONE.

[14] Cheetham M, Wiseman A, Khazaeli B, Gibson E, Gray P, Van der Graaf P (2018). Embedded research: a promising way to create evidence-informed impact in public health?. J Public Health (Oxf).

[15] van der Graaf PFL, Adams J, Shucksmith J, White M (2017). How do public health professionals view and engage with research? A qualitative interview study and stakeholder workshop engaging public health professionals and researchers. BMC Public Health.

[16] The Academy of Medical Sciences (2016). Improving the health of the public by 2040. Optimising the research environment for a healthier, fairer future.

[17] Lockwood A, Walters H (2018). Making the most of public health research. J Public Health (Oxf).

[18] Department of Health (2018). Eligiblity Criteria for NIHR Clinical Research Network Support.

[19] Cheetham M, Redgate S, Van der Graaf P, Hunter R (2019). L. R. Local Authority Champions of Research Project: A Report for the Health Foundation.

[20] Vassilev I, Rogers A, Kennedy A, Oatley C, James E (2019). Identifying the processes of change and engagement from using a social network intervention for people with long-term conditions. A qualitative study. Health Expectations..

[21] McGrath C, Palmgren PJ, Liljedahl M (2018). Twelve tips for conducting qualitative research interviews. Medical Teacher..

[22] Marshall C, Rossman GB (2006). Designing Qualitative Research.

[23] South E, Lorenc T (2020). Use and value of systematic reviews in English local authority public health: a qualitative study. BMC Public Health.

[24] Oliver KA, de Vocht F, Money A, Everett M (2017). Identifying public health policymakers’ sources of information: comparing survey and network analyses. Eur J Public Health.

[25] van der Graaf PBL, Holding E, Goyder E (2021). What makes a successful collaborative reserach project between public health practitioners and academics? A mixed methods review of funding applications submitted to a local evaluation scheme. Health Res Policy Syst.

[26] Kneale D, Rojas-García A, Thomas J (2018). Exploring the importance of evidence in local health and wellbeing strategies. J Public Health (Oxf).

[27] Allen P, Jacob RR, Lakshman M, Best LA, Bass K, Brownson RC (2018). Lessons Learned in Promoting Evidence-Based Public Health: Perspectives from Managers in State Public Health Departments. J Community Health.

[28] Peirson L, Ciliska D, Dobbins M, Mowat D (2012). Building capacity for evidence informed decision making in public health: a case study of organizational change. BMC Public Health.

[29] Masood Sara KA, Regan S (2018). The use of research in public health policy: a systematic review. Evidence & Policy.

[30] van der Graaf PCM, Redgate S, Humble C, Adamson A (2021). Co-prodution in local governement: process, codification and capacity building of new knowledge in collective reflection spaces. Workshops findings from a UK mixed methods study. Health Res Policy Syst.

[31] Tong A, Sainsbury P, Craig J (2007). Consolidated criteria for reporting qualitative research (COREQ): a 32-item checklist for interviews and focus groups. Int J Qual Health Care.

